# Quick Detection of *Proteus* and *Pseudomonas* in Patients’ Urine and Assessing Their Antibiotic Susceptibility Using Infrared Spectroscopy and Machine Learning

**DOI:** 10.3390/s23198132

**Published:** 2023-09-28

**Authors:** George Abu-Aqil, Itshak Lapidot, Ahmad Salman, Mahmoud Huleihel

**Affiliations:** 1 Department of Microbiology, Immunology, and Genetics, Faculty of Health Sciences, Ben-Gurion University of the Negev, Beer-Sheva 84105, Israel; 2 Department of Electrical Engineering, ACLP-Afeka Center for Language Processing, Afeka Tel-Aviv Academic College of Engineering, Tel-Aviv 69107, Israel; itshakl@afeka.ac.il; 3 Laboratoire Informatique d’Avignon (LIA), Avignon Université, 339 Chemin des Meinajaries, 84000 Avignon, France; 4 Department of Physics, SCE-Shamoon College of Engineering, Beer-Sheva 84100, Israel

**Keywords:** urinary tract infection (UTI), *Proteus mirabilis*, *Pseudomonas aeruginosa*, bacterial resistance, infrared spectroscopy, machine learning

## Abstract

Bacterial resistance to antibiotics is a primary global healthcare concern as it hampers the effectiveness of commonly used antibiotics used to treat infectious diseases. The development of bacterial resistance continues to escalate over time. Rapid identification of the infecting bacterium and determination of its antibiotic susceptibility are crucial for optimal treatment and can save lives in many cases. Classical methods for determining bacterial susceptibility take at least 48 h, leading physicians to resort to empirical antibiotic treatment based on their experience. This random and excessive use of antibiotics is one of the most significant drivers of the development of multidrug-resistant (MDR) bacteria, posing a severe threat to global healthcare. To address these challenges, considerable efforts are underway to reduce the testing time of taxonomic classification of the infecting bacterium at the species level and its antibiotic susceptibility determination. Infrared spectroscopy is considered a rapid and reliable method for detecting minor molecular changes in cells. Thus, the main goal of this study was the use of infrared spectroscopy to shorten the identification and the susceptibility testing time of *Proteus mirabilis* and *Pseudomonas aeruginosa* from 48 h to approximately 40 min, directly from patients’ urine samples. It was possible to identify the *Proteus mirabilis* and *Pseudomonas aeruginosa* species with 99% accuracy and, simultaneously, to determine their susceptibility to different antibiotics with an accuracy exceeding 80%.

## 1. Introduction

Antibiotics have been a crucial approach in preventing and treating human bacterial infections for the past century. These powerful medications effectively combat specific diseases and potentially save lives. Antibiotics destroy bacteria or inhibit their reproduction and spread [1]. After the breakthrough discovery of penicillin, a wide array of antibiotics has been subsequently uncovered [2]. Unfortunately, the repeated and improper utilization of antibiotics has expedited the emergence of mutant bacteria resistant to traditional antibiotics [3,4,5,6]. Urinary tract infections (UTIs) represent the most prevalent bacterial infections, impacting approximately 150 million individuals each year [7,8]. Two of the most incident problematic pathogens are *Proteus mirabilis* and *Pseudomonas aeruginosa*. *Proteus mirabilis* belongs to the *Enterobacteriaceae* family of bacilli and is widely recognized as the primary species of *Proteus* responsible for human infections [9]. *Pseudomonas aeruginosa*, a member of the *Pseudomonadaceae* family, is recognized as the predominant opportunistic pathogen causing infections in humans, commonly encountered in clinical settings [10].

*Proteus* is particularly associated with catheter-associated UTIs, wherein infections occur due to urinary catheterization [11,12,13,14,15]. Bacteremia and sepsis cases related to *Proteus* often arise from UTIs and have a substantial mortality rate [16,17,18,19]. Surface organelles of *Proteus*, such as fimbriae and other adhesins, seem to play a crucial role in the development of the infection [19].

Patients who experience frequent catheter blockages have a higher likelihood of being colonized by *Proteus* compared to those without such obstructions [20]. The issue of catheter-related *Proteus* infections has become increasingly concerning, as existing indwelling urethral catheters lack the ability to resist encrustation by this bacterium in laboratory tests [13,21]. Additionally, *Proteus* can invade bladder epithelial cells and produces various cytotoxins that cause damage to the epithelium, resulting in significant histopathological changes [11]. Hence, *Proteus* is strongly linked to complicated UTIs [22].

On the other hand, *Pseudomonas* is renowned for its ability to thrive in low-nutrient environments and its wide distribution [23]. This versatile bacterium can cause infections in various body organs, including the bloodstream, lungs, urinary tract, and other systems [24].

*Proteus* and *Pseudomonas* infections pose significant challenges for treatment due to their multidrug resistance (MDR), including intrinsic and acquired resistance mechanisms [23]. Therefore, accurate detection and determination of antibiotic susceptibility are crucial for selecting appropriate treatment strategies for infections caused by these bacterial species. Hence, timely diagnosis and treatment of these bacterial infections are paramount to prevent complications and mitigate associated risks. Moreover, this will be an important step toward the limitation of the development and spread of MDR bacteria, including Proteus and Pseudomonas [4,6].

The conventional methods employed in healthcare facilities to determine bacterial susceptibility rely on bacterial culturing and utilize the Vitek 2 System and disc diffusion test. These methods typically necessitate a minimum of 48 h to produce results [25,26]. Consequently, physicians often face the temptation to prescribe antibiotics before receiving the susceptibility results from the bacteriology laboratory.

Fourier transform infrared (FTIR) spectroscopy is a fast and robust method capable of detecting subtle molecular alterations within cells [27,28,29], including those linked to the development of antibiotic resistance. Widely employed across multiple disciplines [30,31,32,33,34,35], FTIR has proven effective in identifying various biological samples, including bacteria and cancer cells [31,36,37,38,39].

Our previous research demonstrated that combining FTIR spectroscopy with machine learning algorithms makes it possible to rapidly identify *E. coli* isolates purified directly from patients’ urine and assess their susceptibility to various antibiotics [40,41] within 40 min of receiving the urine samples. Furthermore, in different studies, our group has shown that this approach can significantly reduce the time required to determine the susceptibility of *Proteus* and *Pseudomonas* strains from 24 h to approximately 20 min following the initial culture [42,43]. To the best of our knowledge, this study demonstrates the feasibility of utilizing FTIR spectroscopy and machine learning as a promising duo to detect *Proteus* and *Pseudomonas* samples isolated directly from urine samples of UTI patients and to simultaneously determine their susceptibility to commonly used antibiotics within approximately 40 min compared to at least 48 h of the classical methods. The substantial reduction in the duration of susceptibility testing is poised to bring about a significant breakthrough in the management of UTI disease.

## 2. Methodology

**Figure 1:**
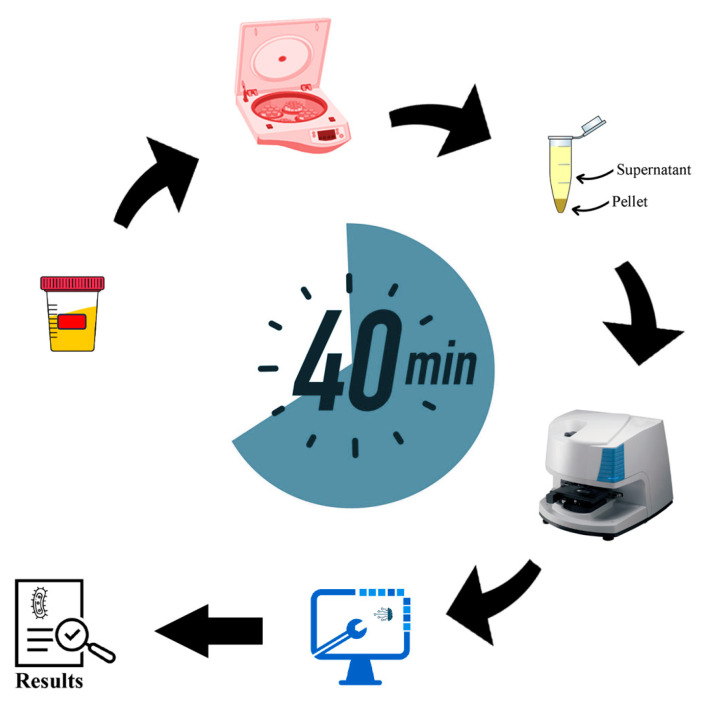
Illustration of the primary steps conducted throughout the current research. Bacterial samples, obtained directly from patients’ urine, were subjected to purification and subsequent measurement via FTIR spectrometry. The resulting spectra were concurrently analyzed for taxonomic classification at the species level and for determining their antibiotic susceptibility.

Figure 1 provides an illustrative depiction of the fundamental stages encompassing the procedures undertaken throughout this study. 

### 2.1. Collection of Bacterial Samples

The bacterial samples analyzed in this study were sourced from the bacteriology lab at Soroka University Medical Center (SUMC). Species identification was performed using Matrix-Assisted Laser Desorption/Ionization Time-of-Flight (MALDI-TOF) technology. For determining the bacterial susceptibility to antibiotics, the Vitek 2 system was utilized. The output from these devices served as the gold standard for this study’s analyses. A total of 3446 patient urine samples were examined, with 360 samples of *Proteus mirabilis*, 353 with *Pseudomonas aeruginosa*, and the remaining samples infected with other UTI bacteria. 

### 2.2. Preparation of Bacterial Samples

Approximately five mL of the patient’s urine was centrifuged at 1000× *g* for five minutes to separate and purify the bacterial contaminants. The resulting pellets were washed three times with double-distilled water (DDW) to remove non-bacterial impurities. The concentration of the bacteria was then assessed by measuring the absorbance at 620 nm using nanodrop, with the bacterial pellets suspended in 50 μL of DDW. Subsequently, the bacterial samples (2 μL containing approximately 5 × 10^5^ cells) were placed on a zinc selenide slide, air-dried at room temperature for approximately five minutes, and analyzed using an FTIR spectrometer.

### 2.3. FTIR Measurements

In this study, a Nicolet-i10 (Thermo Fisher Scientific, Waltham, MA, USA) infrared microscope equipped with a mercury cadmium telluride (MCT) detector cooled by liquid nitrogen was used as the FTIR spectrometer for all experiments. Each bacterial sample extracted from the urine was subjected to 12 measurements taken at different locations of the sample. The measurements were conducted in the 4000–650 cm^−1^ spectral range, employing 128 co-added scans with a spectral resolution of 4 cm^−1^. A background spectrum was recorded from a clean site on the same zinc selenide slide before each sample measurement.

### 2.4. Pre-Processing of the FTIR Measurements

Each measurement underwent multiple pre-processing steps to enhance its quality and extract relevant features [44]. First, atmospheric compensation was applied to eliminate environmental influences. Second, the spectra were smoothed using the Savitzky–Golay algorithm with 13 points to reduce instrumental noise. Third, the spectra were cut to 1800–900 cm^−1^. Fourth, baseline correction was performed using a Concave Rubber Band method with 64 baseline points repeated five times. Fifth, the resulting spectra were further normalized using vector normalization; the absorption intensities at each wavenumber were averaged and then deducted from the initial values (after baseline correction). The resultant vector spectrum had a magnitude of one. Finally, offset correction was applied to shift the intensities of the spectrum minima to zero.

### 2.5. Analysis

This study assessed various machine learning algorithms at each classification level to achieve the most accurate classification results.

## 3. Principal Component Analysis (PCA)

PCA is an algebraic technique used to analyze the variance within a dataset. It accomplishes this by identifying a set of orthonormal vectors, which are the eigenvectors of the covariance matrix, known as principal components (PCs), ordering them in descending order due to their eigenvalues capturing the maximum variance in the data. These PCs are linear combinations of the original vectors. Finally, the original vector is projected on the sub-space spanned by the first eigenvectors, and the resulting coefficients are the new data representations. While PCA is commonly used for dimensionality reduction, it also serves purposes such as data visualization, feature extraction, and noise reduction [45]. This study utilized PCA for data visualization, employing three PCs. The resulting output is a three-dimensional plot that visualizes the data.

## 4. Random Forest (RF)

The RF classifier is an algorithm ensemble method for classification tasks [46]. It combines multiple decision trees to make predictions about the class or category of a given input. Each decision tree in the RF classifier independently classifies the input data based on a subset of features and creates its prediction. Each decision tree is built by randomly selecting a subset of features from the feature vector (in this study, the second derivative of the spectrum) and sampling a subset of training examples. This process is repeated for each individual tree in the ensemble. Each tree is evaluated on a validation set, and only the trees that attain a minimum accuracy of 60% are employed for classification purposes on the test set. The final prediction is determined through majority voting, where each decision tree’s prediction contributes to the result [47,48].

## 5. Validation

**Figure 2:**
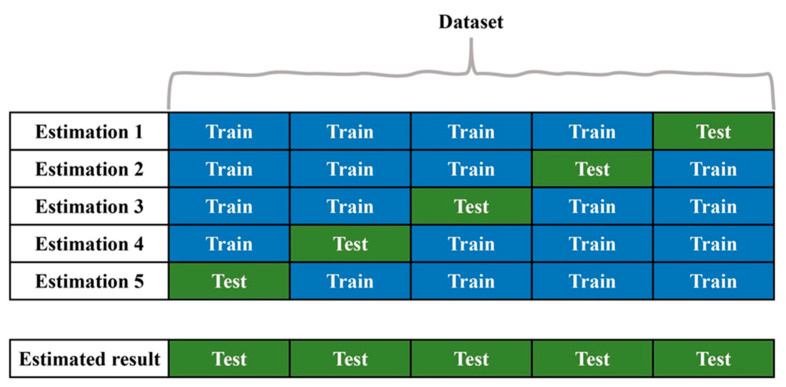
A schematic representation of the machine learning model.

To assess the performance of the RF, K-fold cross-validation was employed, as illustrated in Figure 2. Additionally, nested cross-validation was utilized to optimize its hyperparameters. This technique involves splitting the data into K folds and conducting K iterations, where each fold serves as the evaluation set while the remaining folds act as the training set [49]. The final evaluation score is computed as the accumulation of the decisions obtained from each fold. In this study, we employed K equals five, representing the number of folds used in the cross-validation process. This approach is frequently used when the database is too small. In this way, all the data are used for a test when all the folds are disjointed. The hyperparameter values are chosen via a nested cross-validation procedure and, in the end, are determined by selecting those that yield the highest area under the curve (AUC) of the receiver operating characteristic (ROC). Once selected, the classifier is trained using all the training data with the chosen hyperparameter values. The trained classifier is then used to make predictions on the test data (the remaining fold). In this study, the hyperparameters under consideration were the number of selected features and the depth of the trees. To determine the number of features, the Chi-square technique [50] was employed. This technique involved calculating the Chi-square test value for each wavenumber of the second derivative spectra to evaluate the independence of the category. By calculating the Chi-square statistic for each feature, it becomes possible to rank and select the most relevant features based on their respective values. The number of features considered included nine options ranging from 50 to 400, with a step size of 50 features, in addition to the entire vector comprising 469 features. In terms of the depth of the trees, this study explored six options, ranging from five to ten, with a step size of one. A total of 63 configurations were evaluated to determine the optimal performance of the classifier. The number of trees in the RF ensemble was set at 500. The best configuration was selected based on the highest AUC value of the ROC curve.

## 6. Statistical Parameters

In this study, the sensitive category was characterized as the positive state, while the resistant category was defined as the negative state. The classifier’s performance was assessed based on measures such as accuracy, sensitivity, specificity, positive predicted value (PPV), and negative predicted value (NPV). Sensitivity refers to the probability of correctly identifying positive samples as positive (sensitive samples), whereas specificity represents the probability of correctly identifying negative samples as negative (resistant samples). PPV quantifies the probability of accurately identifying each sensitive sample among the bacteria predicted as sensitive, while NPV quantifies the probability of accurately identifying each resistant sample among the bacteria predicted as resistant.

## 7. Results and Discussion

This study aims to test the potential of FTIR spectroscopic method-based machine learning for detecting UTI bacteria, whether they are *Proteus* or *Pseudomonas,* and determine their susceptibility to antibiotics (ceftazidime, ciprofloxacin, and gentamicin).

## 8. Representative IR Absorption Spectra

**Figure 3:**
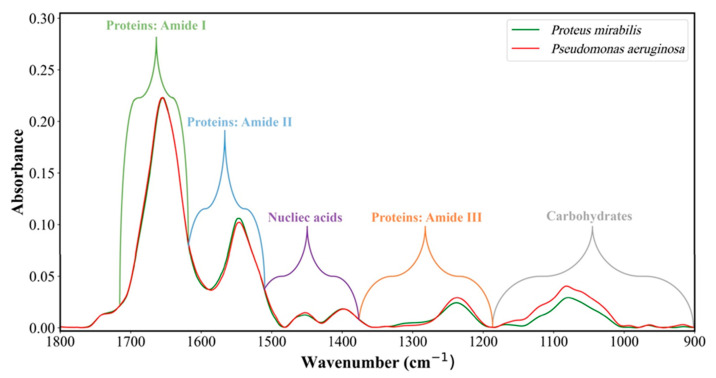
Pre-processed IR absorption spectra of *Proteus* and *Pseudomonas* in the 1800–900 cm^−1^ spectral range. The primary molecules comprising the bacterial samples and their main contribution within the spectrum are labeled.

Figure 3 shows the representative IR absorption spectra of *Proteus* and *Pseudomonas* in the 1800–900 cm^−1^ range. The main absorption bands and corresponding biomolecules of the cells, such as proteins, fatty acids, carbohydrates, and nucleic acids, are labeled.

As shown in Figure 3, each range of the IR absorption spectrum is mainly related to specific biomolecular components of bacterial cells. The various bands arise from distinct modes of vibrations of the functional groups in these biomolecules [37,40,41,51,52,53].

## 9. Identification of *Proteus* and *Pseudomonas* from Other Bacteria

**Figure 4:**
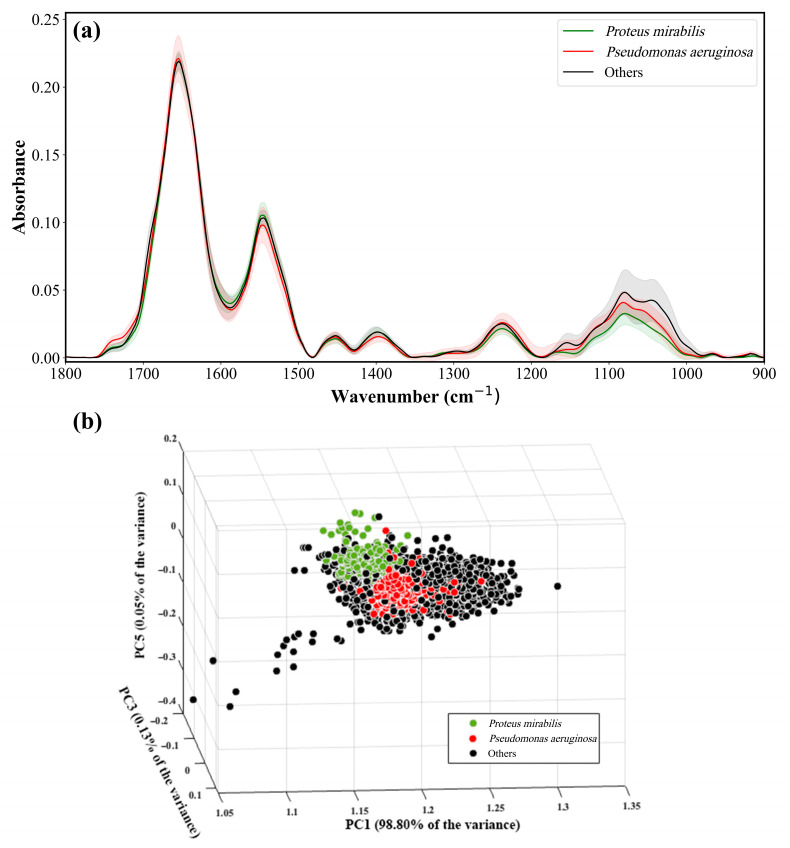
(**a**) Pre-processed IR absorption spectra of *Proteus*, *Pseudomonas*, and other UTI bacteria in the spectral range of 1800–900 cm^−1^. The highlighted area represents the error calculated as standard deviation. (**b**) 3D plot of the scores of PC1, PC3, and PC5 for the three categories of bacteria is shown. Each spectrum is represented as a single point in the plot, and the coordinates of each point are the coefficients of the PCs used to create the plot.

The taxonomic identification of the UTI-infecting bacterium at the species level is the first step in planning appropriate antibiotic treatment. Figure 4a shows the average IR absorption spectra of 360 *Proteus*, 353 *Pseudomonas*, and 2733 other UTI bacterial samples in the 1800–900 cm^−1^ range. The shadows represent errors calculated as the standard deviation. For visualization and to evaluate the complexity of classification, we generated a 3D plot of the spectra of *Proteus*, *Pseudomonas*, and other UTI bacteria based on PCA calculation. PCA is an unsupervised method for dimensionality reduction. PCA transforms the spectra into a new domain, where each spectrum is represented as a linear combination of the new PCs. Figure 4b shows a projection of the spectra of *Proteus*, *Pseudomonas*, and other UTI bacteria data on a 3D subspace spanned by PC1, PC3, and PC5 for visualization. We tried different triples of PCs, and the best plot was chosen in Figure 4b. Each spectrum is represented as a single point in the plot, and the variance of each PC was labeled.

As depicted in Figure 4a, the differences among the individual IR absorption spectra are primarily evident in the intensities. Despite these spectral changes, the IR absorption spectra overlap and are similar. Despite some noticeable distinctions in Figure 4b, there are numerous instances of overlapping points between the different classes. Hence, it is crucial to employ a sophisticated classifier like RF to differentiate among these classes effectively. The performance of the RF classifier is presented in Table 1, demonstrating a confusion matrix with an accuracy rate of ~99%.

Following that, it is necessary to determine the susceptibility of each type of bacteria to various antibiotics using its dataset separately. To achieve this goal, 360 samples of *Proteus* and 353 samples of *Pseudomonas* were analyzed separately to determine their susceptibility to ceftazidime, ciprofloxacin, and gentamicin antibiotics separately.

## 10. Susceptibility Determination of *Proteus* and *Pseudomonas* to Antibiotics

**Figure 5:**
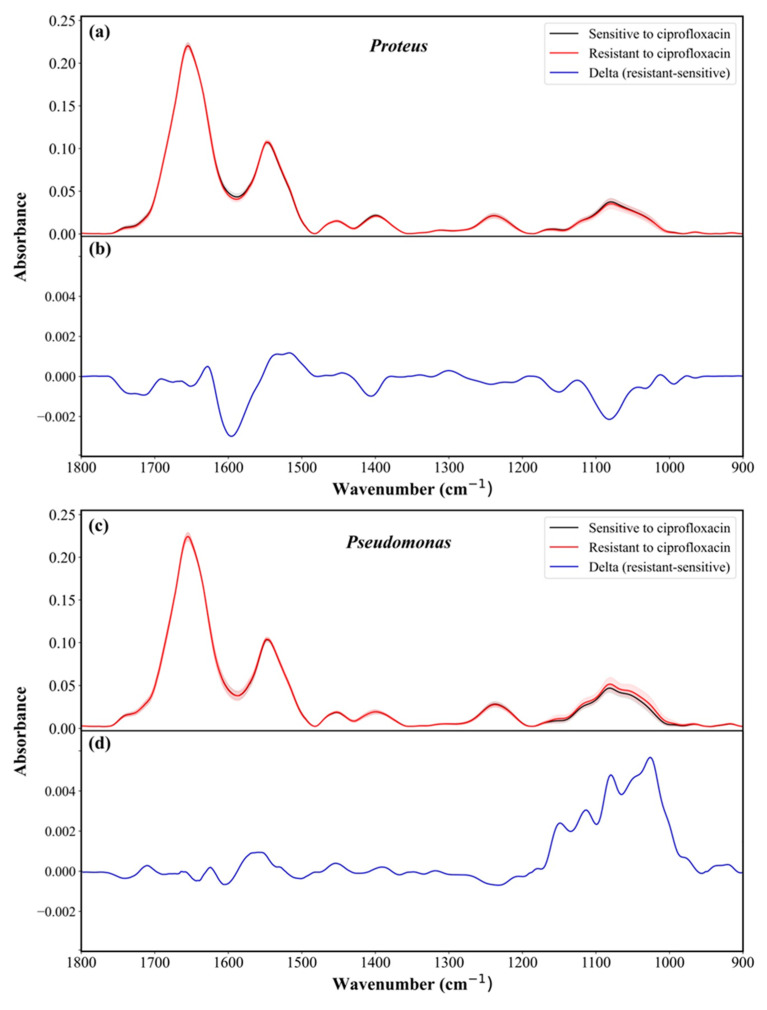
Average sensitive and resistant IR absorption spectra of (**a**) *Proteus* and (**c**) *Pseudomonas* to ciprofloxacin after pre-processing in the 1800–900 cm^−1^ region. The errors were calculated as standard deviation and visualized as a shadow area. The difference spectrum was calculated as the average resistant spectrum minus sensitive IR absorption spectrum of (**b**) *Proteus* and (**d**) *Pseudomonas*.

Figure 5a displays the pre-processed IR absorption spectra of *Proteus*, depicting both sensitive and resistant samples to ciprofloxacin. The shaded region represents the standard deviation. Figure 5b showcases the difference between the average of the resistant and sensitive spectra for the same antibiotic calculated as the average resistant spectrum minus the average sensitive spectrum. Similarly, Figure 5c,d exhibits the corresponding figures for *Pseudomonas*. Comparable plots (Figures S1 and S2) were generated for the remaining investigated antibiotics. As expected, the differences between the spectra are minor since the resistivity is acquired due to a small change in the bacterial cell [54,55]. To acquire high signal-to-noise ratio spectra from each sample, a precise sample preparation procedure was applied as described in our previous studies [40,41,43,54,56]. Furthermore, from each sample, 12 spectra were measured from distinct points within the same sample, further enhancing the accuracy of the utilized classifier through improved reproducibility of the spectra.

**Figure 6:**
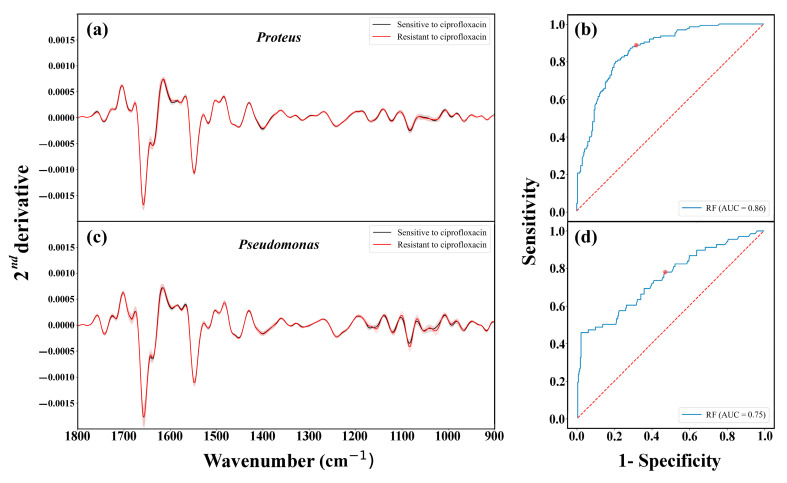
Averages of second derivative IR absorption spectra of *Proteus* (**a**) and *Pseudomonas* (**c**) in the 1800–900 cm^−1^ region, grouped based on their susceptibility to ciprofloxacin antibiotic. The errors were calculated as standard deviation and visualized as a shadow area. (**b**,**d**) represent the ROC curves of the classification. The red dots are operation points at which the RF classifier was run.

The differentiation between the samples based on their susceptibility to the investigated antibiotics using PCA is shown in Figure S3 as a projection of the spectra on a 3D subspace spanned by PC1, PC2, and PC3 for visualization. As can be seen, the data points overlap, and poor differentiation results were obtained (Figure S3). However, these differences are still present and enable the RF classifier to differentiate between the classes successfully. Notably, Figure 5b,d, Figures S1b,d and S2b,d highlight that the main disparities appear in the Amide I, Amide II, and carbohydrates regions. Notably, significant differences between the sensitive and resistant isolates of the same species are observed in the carbohydrate region. Considering the subtle nature of the spectrum variations, the feature vectors for subsequent classification tests were derived as selected features from the second derivative spectra [40,41,43,54,56]. Figure 6a presents the average second derivative IR absorption spectra of sensitive and resistant *Proteus* samples, after pre-processing, to the ciprofloxacin antibiotic. Similarly, Figure 6c showcases the corresponding figure for *Pseudomonas*. The performance of the RF classifier was evaluated as ROC curves in Figure 6b for *Proteus* and Figure 6d for *Pseudomonas*. Similar figures were generated for ceftazidime and gentamicin antibiotics, as shown in Figures S4 and S5. A summary of the RF classifier’s performance for all investigated antibiotics is listed in Table 2.

Based on the findings presented in Figure 6, Figures S4 and S5 and Table 2, the RF classifier has achieved reasonable success rates considering the limited number of samples in the datasets. It is important to recognize that not all spectral variations observed in Figure 4a,c, Figures S1a,c and S2a,c are solely attributed to the development of resistance; some variations stem from taxonomic differences among the investigated bacterial isolates. Although the success rates of determining susceptibility to antibiotics may not be exceptionally high, these findings are promising. We believe that, based on our experience, expanding the dataset size would enable the utilization of more advanced classifiers, thereby enhancing the overall success rates. 

The success rates achieved in this study were determined within approximately 40 min of receiving the patients’ urine samples. 

It is important to note that a recent study has emphasized the use of MALDI-TOF for the rapid determination of the susceptibility of *Staphylococcus aureus* from highly infected patients to antibiotics [57], without the need for culturing, yielding results in an impressive time frame of 60–80 min. Nonetheless, FTIR offers certain advantages compared to MALDI-TOF [55]. The MALDI-TOF technique, relying on protein molecular weight variations, can frequently misidentify bacteria due to random protein weight changes caused by mutations. Bacterial resistance can stem from specific protein mutations [58], but random mutations unrelated to susceptibility may affect MALDI-TOF accuracy. In contrast, FTIR is unaffected by such molecular changes, requiring a lower bacterial concentration [55,59]. Genotypic methods such as RT-PCR, while quick and sensitive in detecting resistance genes, have limitations. They can only identify potential resistance genes, are less effective with latent infections or sparse samples, and may produce false positives due to contamination, necessitating costly reagents and specialized equipment maintenance [60,61,62]. 

The findings of this study can greatly assist physicians in prescribing the most suitable antibiotic for infected patients with either *Proteus* or *Pseudomonas* bacteria.

Given that providing proper treatment to patients is their utmost concern, physicians currently face challenges in managing MDR bacteria when timely and accurate identification of bacterial resistance to antibiotics is not possible due to the lengthy methods currently employed.

We believe that physicians will be encouraged to change their behavior by reducing the testing time for susceptibility, which can be achieved using our approach. 

This study demonstrated the significant potential of using IR spectroscopy in conjunction with machine learning algorithms to detect *Proteus* or *Pseudomonas* bacteria and determine their susceptibility to various antibiotics.

## 11. Conclusions

The combination of IR spectroscopy and RF shows great promise in bacteriology laboratories to directly detect bacteria and determine their antibiotic susceptibility from a patient’s urine within approximately 40 min.

## Figures and Tables

**Table 1:** Performance of the RF classifier presented as a confusion matrix for the taxonomic classification between *Proteus* (360), *Pseudomonas* (353), and other (2733) UTI bacterial samples.

		Prediction		
		*Proteus*	*Pseudomonas*	Others
True	*Proteus*	0.99 (355)	0.01 (4)	0.00 (1)
	*Pseudomonas*	0.00 (1)	1.00 (352)	0.00 (0)
	Others	0.01 (14)	0.00 (6)	0.99 (2713)

**Table 2:** Performances of the RF classifier for the discrimination between the bacterial isolates as resistant or sensitive to the three different antibiotics.

Antibiotic	Bacteria	Sensitive	Resistant	Features	AUC	Accuracy	Sensitivity	Specificity	PPV	NPV
Ceftazidime	*Proteus*	291	69	469	0.80	0.81	0.86	0.63	0.91	0.51
	*Pseudomonas*	287	63	150	0.78	0.72	0.75	0.57	0.89	0.33
Ciprofloxacin	*Proteus*	236	124	200	0.86	0.82	0.88	0.70	0.85	0.75
	*Pseudomonas*	283	68	50	0.75	0.73	0.78	0.53	0.87	0.37
Gentamicin	*Proteus*	284	76	100	0.82	0.82	0.87	0.62	0.90	0.56
	*Pseudomonas*	301	50	300	0.85	0.81	0.84	0.62	0.93	0.39

## Data Availability

All data and codes are available at: https://github.com/Gerogea/PseAe_PrtMi.git (accessed on 17 July 2023).

## Supplementary Materials

The following supporting information can be downloaded at: https://www.mdpi.com/article/10.3390/s23198132/s1, Figure S1: Average sensitive and resistant IR absorption spectra of (a) *Proteus* and (c) *Pseudomonas* to ceftazidime after pre-processing in the 1800–900 cm^−1^ region. The errors were calculated as standard deviation and visualized as a shadow area. The difference spectrum was calculated as average resistant minus average sensitive IR absorption spectra of (b) *Proteus* and (d) *Pseudomonas*. Figure S2: Average sensitive and resistant IR absorption spectra of (a) *Proteus* and (c) *Pseudomonas* to gentamicin after pre-processing in the 1800–900 cm^−1^ region. The errors were calculated as standard deviation and visualized as a shadow area. The difference spectrum was calculated as average resistant minus sensitive IR absorption spectra of (b) *Proteus* and (d) *Pseudomonas*. Figure S3: 3D plot of the scores of PC1, PC2, and PC3 for the two bacteria is shown. The projection of *Proteus* based on the susceptibility to (a) ceftazidime, (c) ciprofloxacin, and (e) gentamicin are presented. Similar plots were generated for *Pseudomonas* regarding the same antibiotics in (b,d,e), respectively. Each spectrum is represented as a single point in the plot, and the coordinates of each point are the coefficients of the PCs used to create the plot. Figure S4: Averages second derivative IR absorption spectra of *Proteus* (a) and *Pseudomonas* (c) in the 1800–900 cm^−1^ region, grouped based on their susceptibility to ceftazidime antibiotic. The errors were calculated as standard deviation and visualized as a shadow area; (b,d) Represent the ROC curves of the classification. Figure S5: Averages second derivative IR absorption spectra of *Proteus* (a) and *Pseudomonas* (c) in the 1800–900 cm^−1^ region, grouped based on their susceptibility to gentamicin antibiotic. The errors were calculated as standard deviation and visualized as a shadow area. (b,d) Represent the ROC curves of the classification.
