# Supplementary material for: Quick Detection of Proteus and Pseudomonas in Patients’ Urine and Assessing Their Antibiotic Susceptibility Using Infrared Spectroscopy and Machine Learning
Source: Sensors (Basel). 2023 Sep 28;23(19):8132. doi: 10.3390/s23198132 (PMC10575053; doi:10.3390/s23198132)

Figure S1: Average sensitive and resistant IR absorption spectra of (a) *Proteus* and (c) *Pseudomonas* to ceftazidime after pre-processing in the 1800-900 cm^{-1} region. The errors were calculated as standard deviation and visualized as a shadow area. The difference spectrum was calculated as average resistant minus average sensitive IR absorption spectra of (b) *Proteus* and (d) *Pseudomonas*.

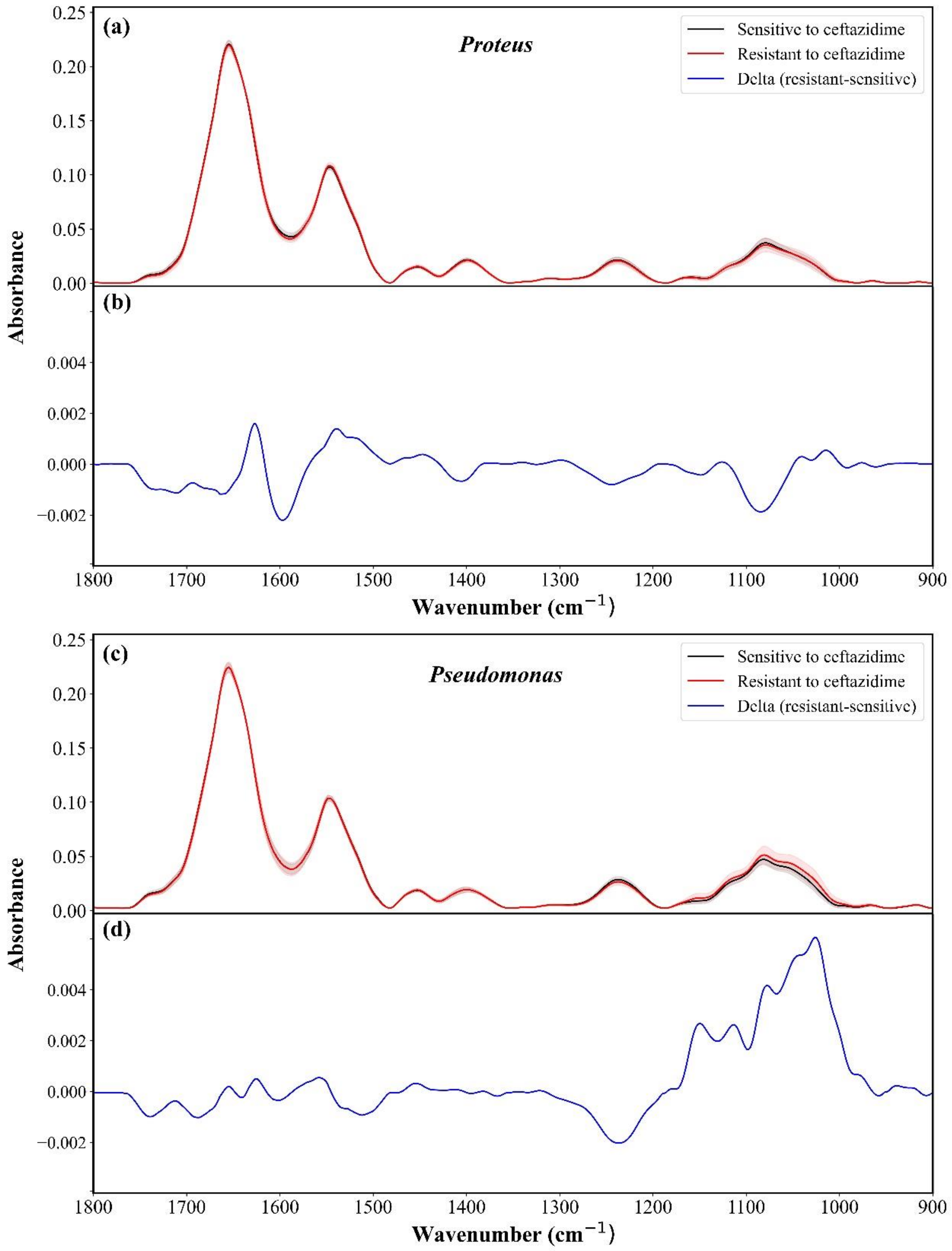

Figure S2: Average sensitive and resistant IR absorption spectra of (a) *Proteus* and (c) *Pseudomonas* to gentamicin after pre-processing in the 1800-900 cm^{-1} region. The errors were calculated as standard deviation and visualized as a shadow area. The difference spectrum was calculated as average resistant minus sensitive IR absorption spectra of (b) *Proteus* and (d) *Pseudomonas*.

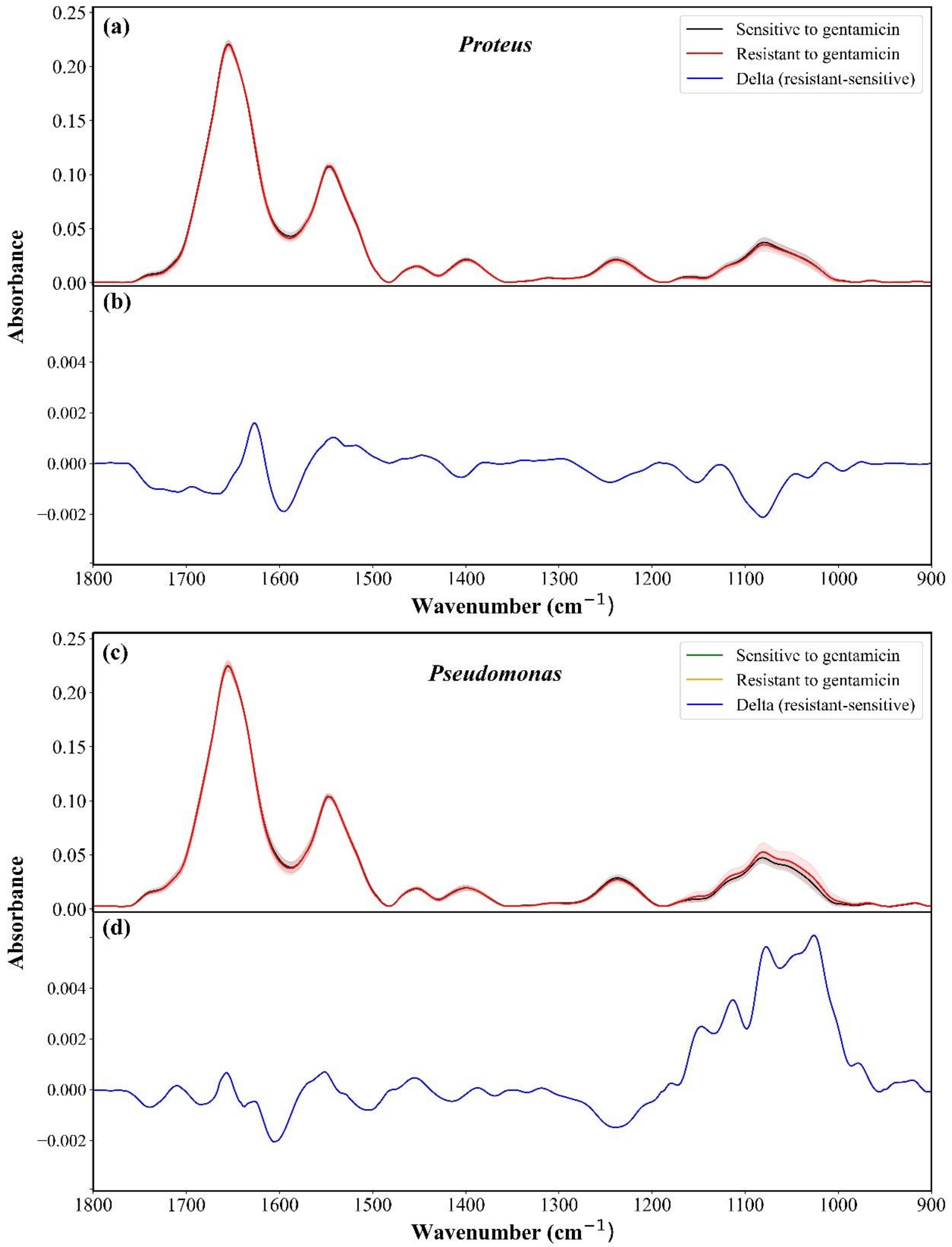

Figure S3: 3D plot of the scores of PC1, PC2, and PC3 for the two bacteria is shown. The projection of *Proteus* based on the susceptibility to (a) ceftazidime, (c) ciprofloxacin, and (e) gentamicin are presented. Similar plots were generated for *Pseudomonas* regarding the same antibiotics in (b), (d), and (e), respectively. Each spectrum is represented as a single point in the plot, and the coordinates of each point are the coefficients of the PCs used to create the plot.

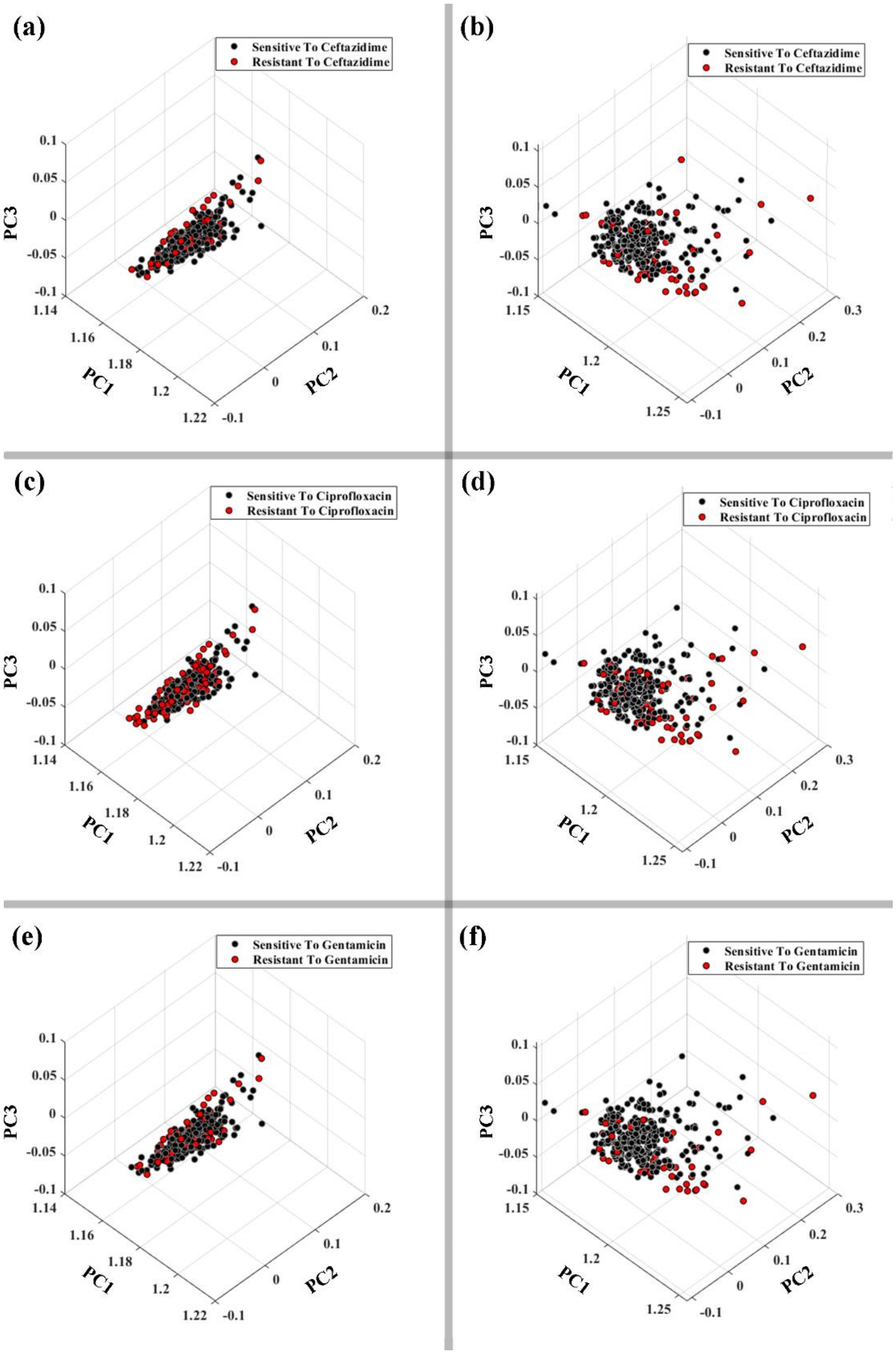

Figure S4: Averages second derivative IR absorption spectra of *Proteus* (a) and *Pseudomonas* (c) in the 1800-900 cm^{-1} region, grouped based on their susceptibility to ceftazidime antibiotic. The errors were calculated as standard deviation and visualized as a shadow area. Figures S4 (b), and (d) represent the ROC curves of the classification.

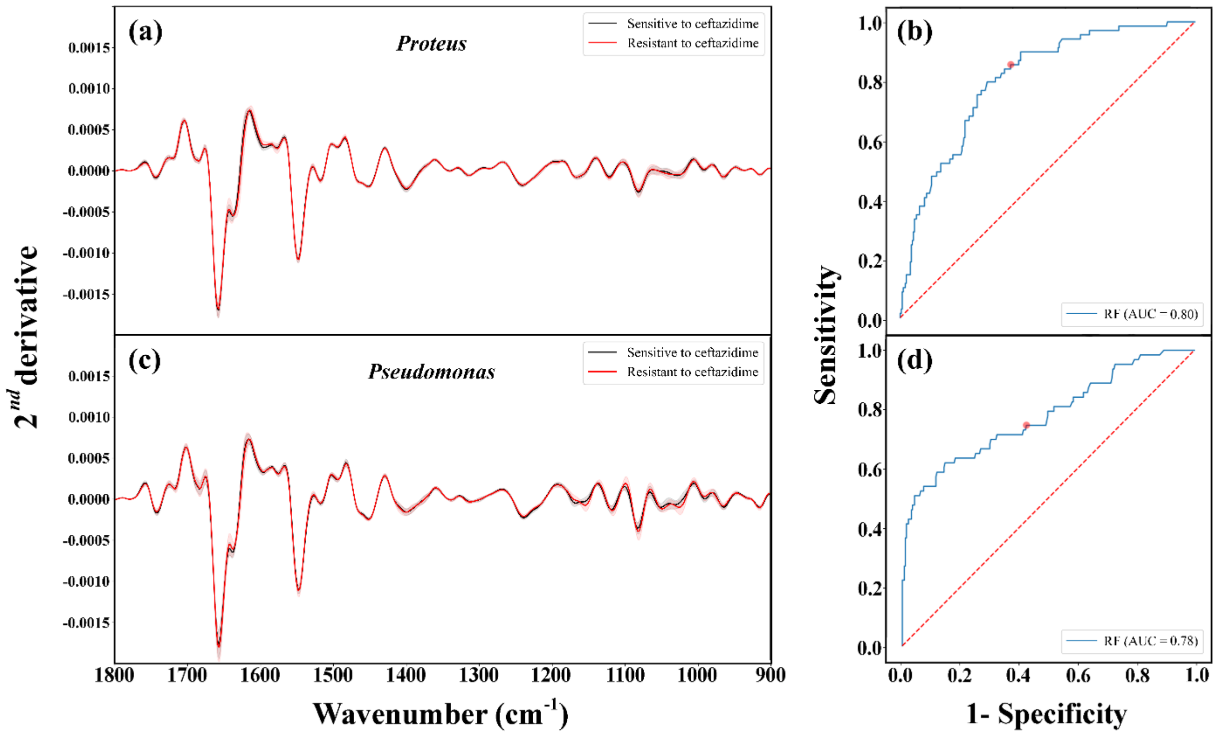

Figure S5: Averages second derivative IR absorption spectra of *Proteus* (a) and *Pseudomonas* (c) in the 1800-900 cm^{-1} region, grouped based on their susceptibility to gentamicin antibiotic. The errors were calculated as standard deviation and visualized as a shadow area. Figures S5 (b), and (d) represent the ROC curves of the classification.

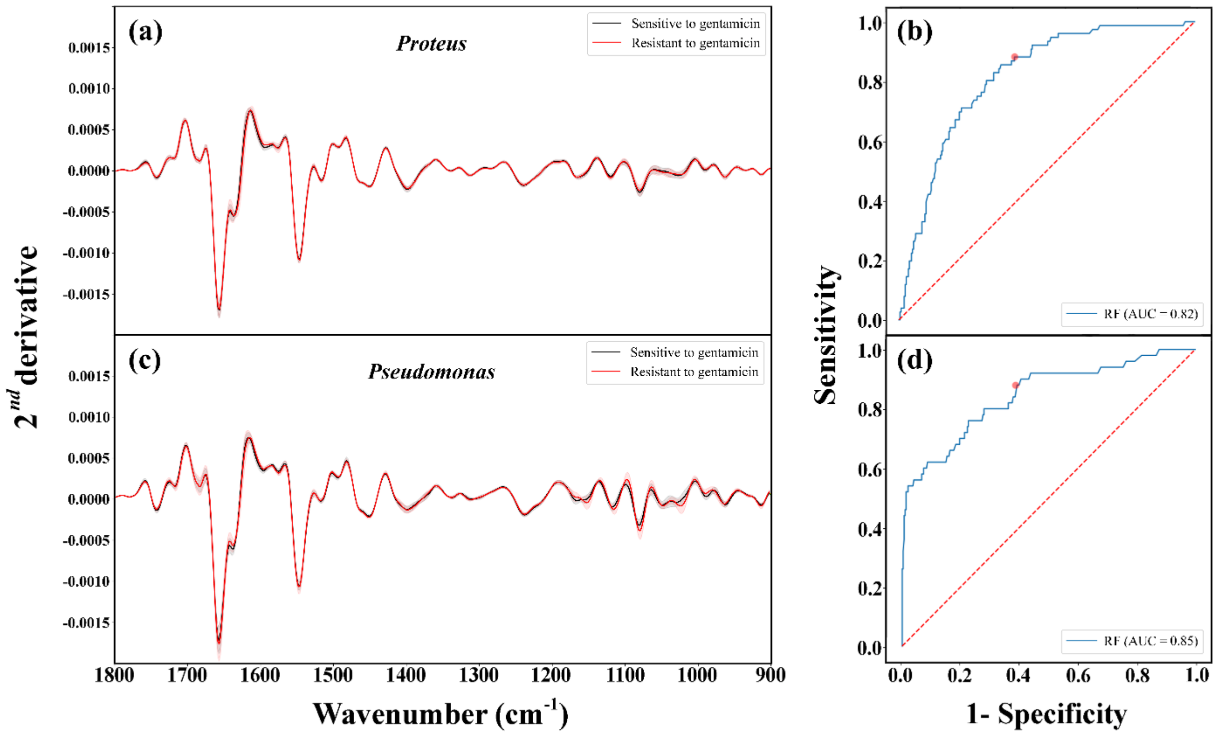

Supplement: Supplementary file 1 [file sensors-23-08132-s001.zip › sensors-2579501-supplementary.pdf]
